# The dengue virus NS1 protein alters *Aedes aegypti* midgut permeability and favors virus dissemination

**DOI:** 10.1128/mbio.03173-25

**Published:** 2026-01-13

**Authors:** Edgar Quezada-Ruiz, Angélica Silva-Olivares, Daniel Talamás-Lara, Grecia Diaz, Sofía Alanís-Muñoz, César A. Pacheco, Ángel Tello-López, Raymundo Cruz, Abigail Betanzos, Salvador Hernández-Martínez, Humberto Lanz-Mendoza, Juan E. Ludert

**Affiliations:** 1 Department of Infectomics and Molecular Pathogenesis, Center for Research and Advanced Studies (Cinvestav), Mexico City, Mexico; 2 National Institute of Public Health, Center for Research on Infectious Diseases, Cuernavaca, Morelos, Mexico; 3 Electron Microscopy Unit (LANSE), Center for Research and Advanced Studies (Cinvestav), Mexico City, Mexico; Tsinghua University, Beijing, China

**Keywords:** dengue virus, NS1 protein, midgut permeability, septate junctions, *Aedes aegypti*, dissemination, vectorial competence

## Abstract

The dengue virus is an arbovirus of public health importance transmitted by *Aedes aegypti* mosquitoes. The NS1 protein is a multifunctional glycoprotein, highly conserved among Orthoflaviviruses, that is secreted from infected cells, circulates in the sera of dengue patients at high concentrations, and has been involved with pathogenesis by several mechanisms, including vascular leakage and immune evasion. However, the role of NS1 within the mosquito vector is not fully understood. In this study, we observed that feeding female *Aedes aegypti* mosquitoes with blood meals supplemented with recombinant NS1 resulted in increased midgut permeability, as evaluated by the diffusion of the non-toxic dye Brilliant Blue FCF into the hemocoel. Histological and transmission electron microscopy analyses revealed epithelial damage and disruption of the midgut cellular junctions. Immunofluorescence assays further demonstrated the delocalization of septate junction proteins essential for epithelial integrity. In addition, metalloproteinase expression in the midgut was also reduced. All these effects were abolished when NS1 was heat-inactivated, indicating that they were NS1-dependent. Significantly, infected blood meals resulted in enhanced dissemination of DENV to secondary organs and earlier virus presence in the mosquito salivary glands, in comparison with meals treated with specific NS1 antibodies. Our findings reveal a novel function for NS1 in mosquitoes and expand the understanding of NS1 functions beyond its human pathophysiological role. In addition, our findings highlight NS1 as a strategic intervention point to reduce transmission efficiency in mosquitoes.

**IMPORTANCE:**

Dengue is the most important viral disease transmitted by mosquitoes to humans. Thus, a deeper understanding of the virus-vector interaction is required for the development of control measures and the mosquito transmission capacity. In this work, we present evidence indicating that the NS1 protein plays a role in the establishment and dissemination of the dengue virus infection in *Aedes aegypti* mosquitoes. Ingested NS1 was found to disrupt the septate junctions and to inhibit metalloproteinase expression of the midgut, allowing the virus to escape the digestive tract. In addition, NS1 was found to promote virus dissemination into the hemocoel, carcass, and salivary glands. These findings uncover new functions for NS1 in the mosquito and highlight ways to interfere with vector competence.

## INTRODUCTION

Dengue fever is the most frequent mosquito-borne viral disease affecting humans and poses a significant socioeconomic burden, particularly in tropical and subtropical regions of the planet (1). Infection may be asymptomatic or present clinical symptoms ranging from a mild fever to high fever, headache, retro-orbital pain, muscle and joint pain, and skin rash. In some cases, dengue fever can progress to severe dengue, characterized by respiratory distress, plasma leakage, severe bleeding, and organ dysfunction (2). Each year, an estimated 390 million infections occur globally, of which approximately 100 million are symptomatic, and 25,000 result in death (3).

The dengue virus (DENV) is an enveloped virus of approximately 50 nm in diameter, belonging to the family *Flaviviridae*, genus *Orthoflavivirus,* and exists as four antigenically distinct serotypes (DENV1–4) (2). The viral genome consists of a single-stranded, positive-sense RNA approximately 10.8 kb in length that encodes a single polyprotein that is cleaved to generate three structural proteins (C, M, and E) and seven nonstructural proteins (NS1, NS2A, NS2B, NS3, NS4A, NS4B, and NS5) (4). The NS1 is a multifunctional protein located in the lumen of the endoplasmic reticulum, as part of the viral replication complexes, but is also secreted along with the virions (5). Circulating NS1 has been linked to pathogenesis by several mechanisms, including complement fixation, generation of host cross-reactive antibodies, induction of pro-inflammatory cytokines, and alteration of vascular homeostasis (6–8). Actually, the circulating NS1 protein of several orthoflaviviruses increases the expression and release of heparanases and sialidases by endothelial cells. When these enzymes are released, they cause degradation of the endothelial glycocalyx, which may contribute to the severe manifestations of the disease, such as plasma leakage (9, 10). In addition, it has been reported that the NS1 of DENV causes the delocalization and phosphorylation of endothelial cell tight junction proteins, such as ZO-1, β-catenin, and VE-cadherin (11), thereby increasing vascular permeability.

DENV is transmitted primarily by *Aedes aegypti* and *Aedes albopictus* (12). When feeding on an infected host, the mosquito acquires the NS1 protein in conjunction with the virion. In the mosquito midgut, the virus undergoes replication and subsequently disseminates to secondary tissues, such as fat body and hemolymph, finally reaching the salivary glands (13). This replicative process in the mosquito is known as the extrinsic incubation period and typically lasts 8–12 days (14, 15). In the mosquito digestive tract, essential physiological events such as protein and nutrient absorption occur in the midgut (15), which is composed of a single monolayer of epithelial cells and a basal lamina (15, 16). While absorption is carried out by the epithelial cells, the basal lamina, composed mainly of laminin and collagen IV, constitutes an impermeable barrier so tight that it prevents the passage of particles larger than 10 nm (17, 18). It is known that the distension of the midgut due to blood feeding causes tissue damage (19–21) and microfractures in the cell monolayer and basal lamina that create escape routes for viruses toward the hemocoel (19, 22). Also, activation of metalloproteinases and other enzymes involved in repairing the basal lamina by pathogen exposure may result in weakening of the lamina (23). However, the mechanisms used by DENV and other orthoflaviviruses to escape the midgut are not fully understood. It has been shown that the DENV NS1 protein can downregulate the transcription of key immune response genes in the mosquito, facilitating infection in the midgut (24). Other factors favoring DENV infection in mosquitoes include gut microbiota (25), host serum iron deficiency (26), and sfRNAs (27). However, it is unknown whether the reported ability of NS1 to permeabilize host endothelia also occurs in the intestinal epithelium of the vector. In this study, we investigate the role of DENV NS1 in altering *Ae. aegypti* midgut permeability and its role in virus dissemination and implications for vectorial competence.

## MATERIALS AND METHODS

### Mosquito rearing

Adult *Ae. aegypti* Rockefeller strain mosquitoes were reared in the insectary of Instituto Nacional de Salud Pública (INSP), Mexico, under the following conditions: 60%–80% humidity, 28°C–30°C, and a 12:12-h light-dark cycle. The larvae were fed with tropical fish food (TetraMin flake fish food). The adult stage was maintained in cages, with *ad libitum* access to a 10% sugar solution in a soaked cotton pad. Adult females aged 5–7 days were used for experiments.

### Organ dissection

Mosquitoes were anesthetized in ice at 4°C and transferred to slides with a drop of phosphate-buffered saline (PBS; NaCl 137 mM, KCl 2.7 mM, Na_2_HPO_4_ 10 mM, and KH_2_PO_4_ 1.8 mM). Dissection was performed using a stereoscopic microscope.

#### Midgut

A transverse cut was made longitudinally from the head to the antepenultimate abdominal segment. The mosquito’s body was separated from the abdomen using forceps, and the midgut was removed intact. Ingested blood was removed by washing several times in PBS, then a pool of five clean guts was placed in microcentrifuge tubes containing 50 µL of PBS.

#### Carcass

Carcasses were recovered after removing the midguts as described above and placed in microcentrifuge tubes containing 50 µL of PBS. All samples were stored at −70°C until used.

### Cell cultures and virus propagation

Baby hamster kidney cells (BHK-21, ATCC CCL-10) were cultured on DMEM supplemented with 5% heat-inactivated fetal bovine serum (FBS; Gibco) under standard conditions of 37°C with 5% CO2. DENV-2 strain New Guinea C was propagated in BHK cells using neonatal mouse brain extract with an MOI of 0.1 in DMEM without FBS. After 5 days post-infection, the supernatants were collected and clarified by centrifugation at 12,000 × *g* for 15 min at 4°C.

The virus was quantified by two-step qPCR using the reagent Maxima SYBR Green/ROX qPCR master mix (2×) (Cat. no. K0221) on a Rotor-Gene 5Q (Qiagen). 800 ng of total RNA was used as a template. For cDNA generation, 1 µL of random hexamer primer (0.2 µg/µL; Cat. no. SO142), 1 µL of dNTPs (10 mM; Cat. no. R0192), and 1 µL of RevertAid (200 U/µL; Cat. no. EP0441) were used in a final volume of 20 µL. DENV primers were used for the mix at a final concentration of 0.3 µM. The reaction parameters were as follows: 10 min at 95°C, and a 40-cycle PCR with the following settings: 95°C for 20 s and 60°C for 45 s. A standard curve using a 10-fold serial dilution of a synthetic gene containing the primer targets flanking regions (gBlock) was used for absolute quantification (28). The Ct values obtained were extrapolated from a standard curve created using the performed dilutions. The concentration obtained was approximately 1 × 10^7^ viral RNA copies/mL. The sequence of the primers and the gBlock are found in Table S1.

### Mosquito feeding with recombinant NS1

Adult female mosquitoes were deprived of sugar for 12 h to facilitate treatment administration. Mosquitoes were fed with 1 mL of rabbit blood containing heparin (250 µL/L) and DMEM (1:1 [vol/vol]) by using a Parafilm membrane artificial feeder, attached to a circulatory water bath at 37°C. Experimental treatments were as follows: (i) negative control, blood + DMEM; (ii) positive control, blood + DMEM + 2.5 mM dithiothreitol (DTT); (iii) recombinant NS1, blood + DMEM + 1 µg recombinant NS1 from DENV-2 (rNS1) produced in HEK293 cells (Biotechne, Cat. No. 9439-DG-100); and (iv) recombinant NS1 heat-denatured, blood + DMEM + 1 µg rNS1 heat-denatured (boiled in a water bath for 30 min). Characterization of the commercial rNS1 using non-denaturing polyacrylamide gel electrophoresis indicated that the NS1 is pure and mainly hexameric in nature (Fig. S1).

**Fig 1:**
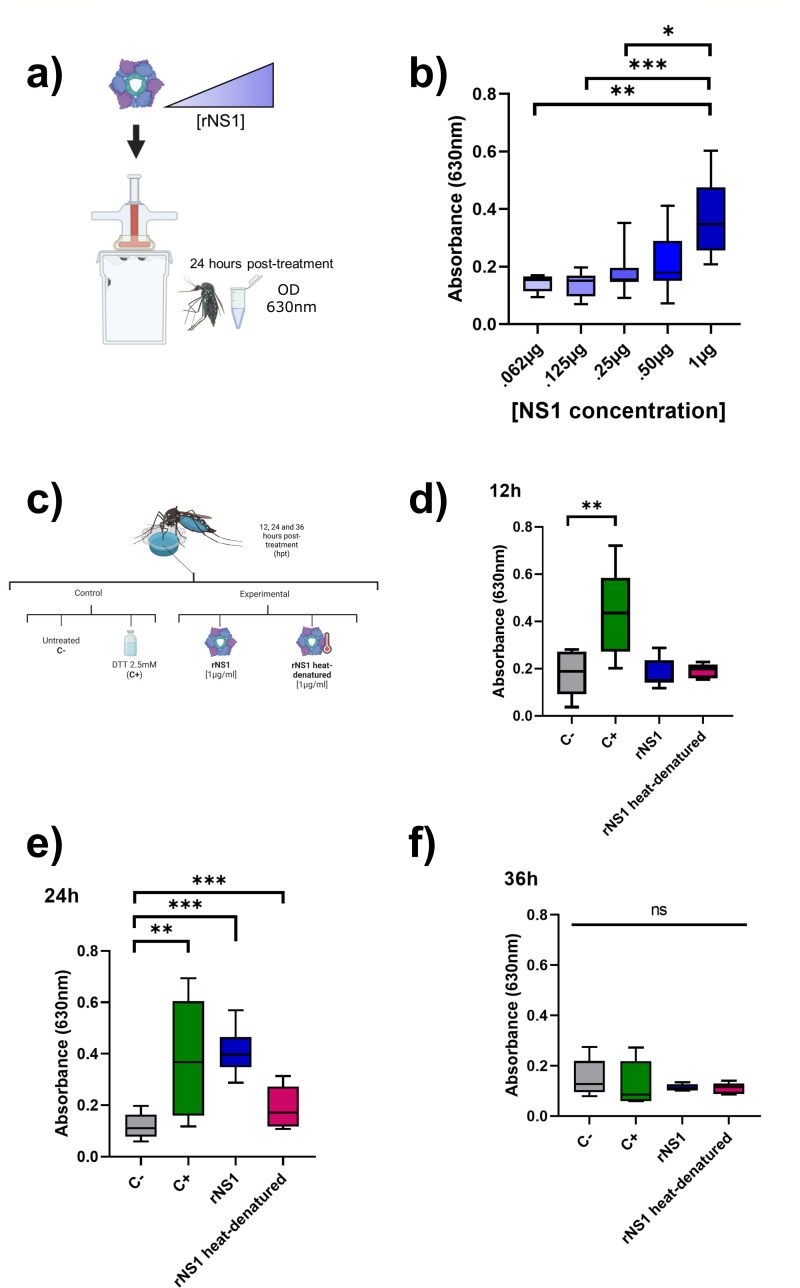
The presence of recombinant NS1 (rNS1) favors the transient outflow of the dye into the hemocoel. (**a**) Schematic representation of the experimental design for the dose-response assay with rNS1. Recombinant NS1 at increasing concentrations was mixed with heparinized rabbit blood and Brilliant Blue FCF and fed to mosquitoes starved for 6 h. At 24 h post-treatment (hpt), mosquitoes were anesthetized, dissected, and the hemolymph collected to read absorbance at 630 nm. (**b**) Dose-dependent increase of midgut permeability observed in response to rNS1: OD measure of Brilliant Blue FCF presence in hemolymph. (**c**) Experimental conditions used to evaluate the role of native and denatured NS1 in modulating epithelial permeability, including untreated (C−), DTT 2.5 mM (C+), recombinant NS1 (rNS1), and heat-denatured rNS1. Quantification of dye outflow at 12 (**d**), 24 (**e**), and 36 (**f**) hpt. rNS1 significantly increased permeability compared to controls at 24 hpt, while heat-denatured rNS1 did not induce a significant effect. No dye leakage was observed at 12 and 36 hpt. Three biological replicates per assay were performed (30 mosquitoes per experiment, *n* = 90 in total). ANOVA/Tukey *P < 0.05; **P < 0.01; ***P < 0.001. ns, no significant difference.

### Intestinal permeability assays

A modified version of the “Smurf assay” (29, 30) was used to assess changes in midgut epithelial permeability. Brilliant Blue FCF dye (Merck, Cat. no. 80717) was prepared at 1% in PBS. Twelve hours before the assay, female mosquitoes were fed with a 10% sucrose solution supplemented with 1% dye. The next day, mosquitoes were starved for 6 h and then fed with rabbit blood mixed with the dye. Hemolymph was collected from mosquitoes exhibiting a blue coloration in their legs (indicative of dye leakage into the hemocoel). The traces of dye that reached the coelomic cavity were recovered by injecting PBS into the thorax region using a microinjector (Nanoject I; Drummond Scientific). Immediately, an incision was made in the last segment, and the PBS/hemolymph that spilled was collected with a micropipette. The hemolymph was clarified by centrifugation at 12,000 × *g* for 10 min to remove hemocytes and fat bodies, and absorbance at 630 nm was measured using a spectrometer (Elisa Plate Reader, DAS, Italy). Values were expressed in relative units. Dye outflow was assessed at 12, 24, and 36 h after dye feeding or post-treatment (hpt). Three biologically independent experiments were performed, and triplicate samples were collected for each treatment.

### Hematoxylin-eosin stains

Samples were processed for histological analysis as described previously (31). Briefly, 24 h after administration of the experimental treatments, abdomens from fully fed adult females were dissected, fixed in Bouin’s solution (5% acetic acid, 9% formaldehyde, 0.9% picric acid, all from Sigma-Aldrich) for 10 min at 56°C, and kept overnight at room temperature (RT). The next day, samples were transferred to 70% ethanol before being embedded in paraffin. Samples were dehydrated in an ethanol-xylene lane (50%, 70%, 80% 95%, and 100% xylene) and embedded in Paraplast X-TRA (Oxford, St. Louis, MO) overnight at 56°C. Abdomens were mounted in Paraplast X-Tra using histological cassettes (Quebec, Canada). Six-micrometer-thick histological sections were made in a longitudinal plane and mounted on slides pre-treated with 1% gelatin (Sigma-Aldrich, St. Louis, MO) (31). After paraffin removal, slides were hydrated and stained using the standard aqueous Harris hematoxylin-eosin protocol. Five female mosquitoes’ abdomens per treatment were examined with three biological replicates.

### Transmission electron microscopy

#### Sample preparation and paracellular permeability assay

Five midguts per treatment were dissected as described above, fixed with 2.5% glutaraldehyde dissolved in 0.1 M sodium cacodylate buffer, pH 7.2, at RT, and post-fixed with 1% osmium tetroxide. After dehydration in increasing concentrations of alcohol, the samples were pre-embedded in a mixture of 100% ethanol and Polybed resin (2:1 and 1:1), before embedding in pure resin. The resin was left to polymerize at 60°C for 24 h. Subsequently, 60 nm thin sections were made and contrasted with uranyl acetate and lead citrate. To visualize the septate junctions (SJs), sections were further stained with 0.6% ruthenium red, as described (32, 33), and finally analyzed with a JEOL-JEM-1400 transmission electron microscope (TEM; JEOL Ltd., Tokyo, Japan).

### Localization of SJ proteins

To evaluate changes in the localization of proteins involved in the SJs, female abdomens collected 24 hpt were dissected as described above and fixed for 2 h at RT in 4% paraformaldehyde in PBS, pH 7.2. After three washes with PBS, the abdomens were placed in 10% sucrose overnight. Samples were embedded in tissue freezing medium (Leica Microsystems, Wetzlar, DE) and immediately frozen at −20°C.

The embedded abdomens were cryosectioned in the transversal plane by using a cryostat (Leica CM1100, Leica Microsystems). In the midgut region, serial sections of 6 µm thick sections were obtained and attached to slides pretreated with 0.5% gelatin (Sigma) and potassium dichromate (Merck) 0.05%. Sections were permeabilized with methanol at −20°C for 7 min, followed by blocking with 1% bovine serum albumin in PBS containing 0.1% Tween-20 (PBS-Tw). A rabbit anti-claudin-1 Mab (1:100; Invitrogen, cat. no. 51900) and mouse anti-β-catenin Mab (1:50; Santa Cruz cat. no. sc376959) were used as primary antibodies. Samples were incubated with primary antibodies at 4°C overnight. After three washes with PBS, samples were incubated for 2 h at RT, with a goat anti-rabbit AlexaFluor 488 and a mouse anti-IgG AlexaFluor 647 (Invitrogen), respectively. Nuclei were stained with a DAPI solution (1:800; Sigma D9542) for 10 min. Samples on the slides were mounted in 5 µL of VectaShield (Vector, H-1000) and analyzed with an LSM 900 confocal microscope. Images were processed with Zeiss Zen Blue 3.6 software. Three abdomens of female mosquitoes per treatment were examined.

### Analysis of the dissemination of DENV to secondary tissues

To evaluate the role of NS1 on virus dissemination, DENV-2 supernatants from BHK-21 cells were incubated for 1 h at RT either with purified rabbit pre-immune serum at a concentration of 8 µg of IgG (1:100) or purified rabbit polyclonal anti-NS1 at a concentration of 9 µg of IgG (1:100). Sera were in-house made, purified by affinity chromatography (HiTrap Protein G HP, Cytiva, cat # 29048581), and have been previously tested for DENV NS1 specificity (34). Then, DENV-2 samples were mixed with fresh rabbit blood (1:1), and mosquitoes were fed using a Parafilm membrane artificial feeder. Infection was evaluated at 24, 48, and 72 h post-feeding in the midgut and carcass. In addition, mosquitoes’ saliva was obtained at 7 dpi to evaluate the presence of viral RNA (35). Total RNA was extracted and reverse transcribed as described above. PCRs were performed using 100 ng of cDNA as a template. The primers used are described in Table S1. Relative quantification was performed using the endogenous gene *eEF1α* with the Pfaffl method (36). The infection ratio was calculated by dividing the number of positive samples obtained by RT-qPCR for DENV-2 by the total number of samples analyzed by tissue and time post-infection, expressing the results as a percentage. Three biological replicates were made, collecting five pools of five tissues per replicate.

### Statistical analysis

A normality test was performed for each data set before employing any statistical test. For data with a normal distribution, a one-way ANOVA Tukey test was used, and for non-normally distributed data, a nonparametric test (one-way ANOVA/Kruskal-Wallis test) was used. Infection prevalence was compared using a Fisher’s exact test. A two-way ANOVA/Bonferroni’s multiple comparisons test was used to compare the spread of the virus to secondary tissues. The criteria used were *P < 0.5, **P < 0.05, ***P < 0.001. All analyses were performed, and graphs were created using GraphPad Prism 8.0.2 statistical software (37).

## RESULTS

### NS1 increases midgut epithelial permeability in *Ae. aegypti*

To determine whether the NS1 protein would affect midgut permeability, the outflow of blue dye was quantified in mosquitoes fed with rNS1. Initially, a dose-response assay was performed using increasing concentrations (0.062–1.0 µg) of rNS1, and collecting the samples 24 hpt (Fig. 1a). Although a plateau was not reached, the results showed a clear dose-dependent effect, where 1 µg/mL produced the most significant dye leakage into the hemocoel (Fig. 1b). This concentration of rNS1 was used in a time course carried out at 12, 24, and 36 hpt. Heat-inactivated rNS1 and an untreated condition were used as negative controls, and DTT as a positive control. Mosquitoes fed with DTT showed a significant outflow of the dye at 12 and 24 hpt (Fig. 1d and e), indicating disturbance in the midgut epithelium, probably due to disruption of the disulfide bonds that stabilize the proteins in the SJs (18). Interestingly, rNS1 produced dye outflow into the hemocoel at 24 hpt, similar to the ones observed with DTT (C−; *P* < 0.01). This effect was not observed with heat-denatured rNS1 (*P* < 0.05). The changes caused by the rNS1 at 24 hpt were not observed at 12 hpt, suggesting a delay in relation to DTT-induced changes. At 36 hpt, no changes were observed with any treatment, suggesting a repair of the damage process (Fig. 1f). All these results indicate that NS1 produces diffusion alterations and midgut barrier dysfunction in mosquitoes. To evaluate whether the observed changes were specific to NS1 of DENV, rNS1 from ZIKV was also tested. The results showed a clear tendency (*P* = 0.06) of dye outflow with rNS1, which was fully reversed when the rNS1 was heat denatured (Fig. S1 and S2). These results suggest that ZIKV rNS1 also induces intestinal midgut permeability when fed to mosquitoes.

### NS1 induces histological changes in the mosquito midgut

Histological analysis of the midgut of the mosquitoes exposed to rNS1 was performed on longitudinal sections of the abdomen. Previously, to establish the half-life of the rNS1 in the midgut, dot-blot assays were carried out. NS1 was observed in the midguts at 24 hpt, but not at 48 hpt, suggesting exit or degradation of NS1 (Fig. S3). Thus, the following experiments were all performed at 24 hpt.

**Fig 2:**
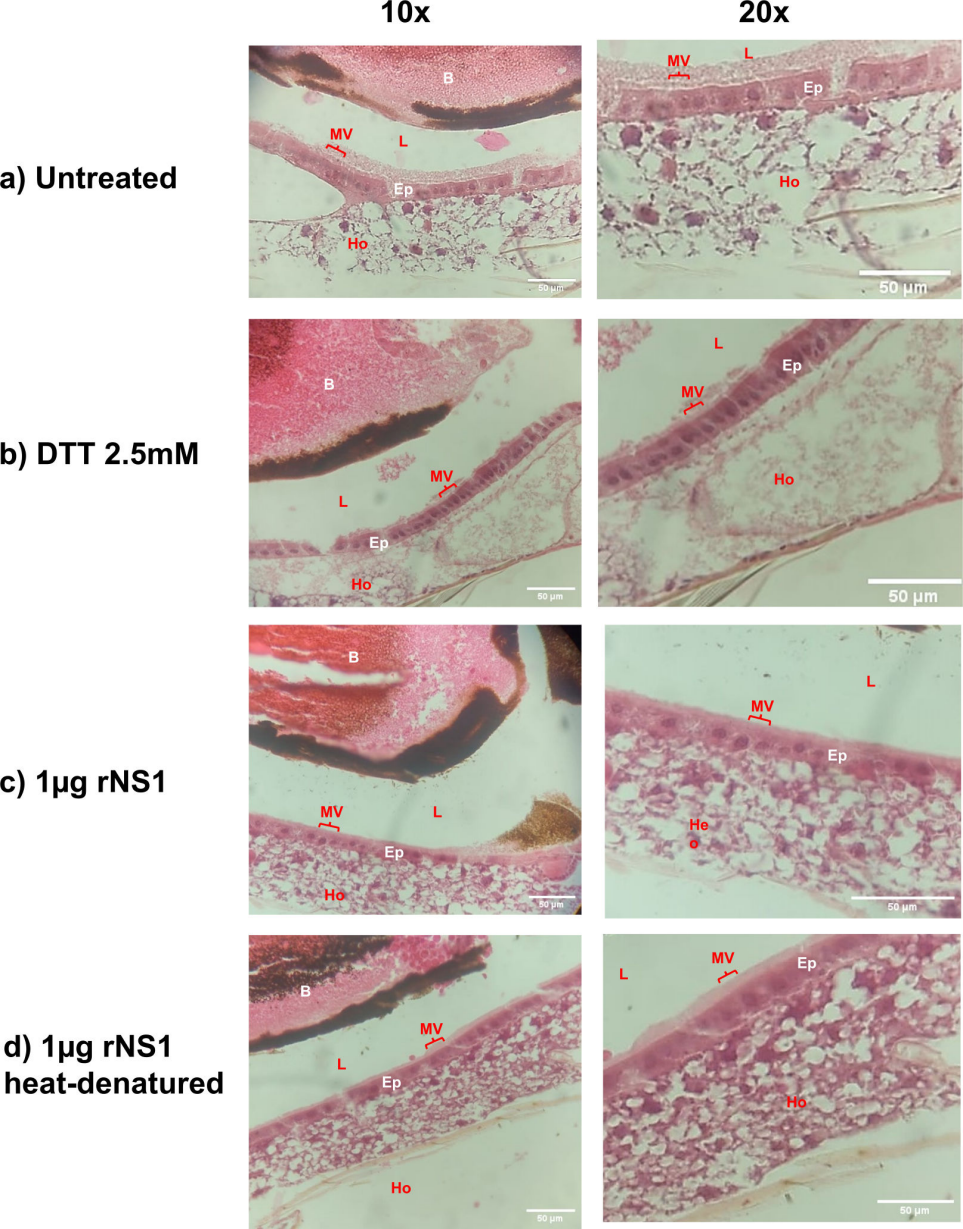
Reduction of microvilli in midguts of *Aedes aegypti* exposed to rNS1 protein. Longitudinal views of H-E-stained sections of the epithelial midgut. Structural changes were observed in the zone of microvilli when rNS1 was present (**c**). There was no reduction in microvilli when rNS1 was heat denatured (**d**), absent (**a**), or treated with DTT (**b**). Three abdomens of female mosquitoes per treatment were examined with reproducible results. Lumen (L), epithelial cells (Ep), microvilli (MV), and hemocoel (Ho). Scale bar: 50 µm.

Hematoxylin-eosin staining showed a loss in the microvilli of epithelial cells in the midguts of the mosquitoes fed with blood and rNS1 protein when compared to the control, untreated condition (Fig. 2a through c). This effect was not observed when heat-denatured rNS1 was used (Fig. 2d). No evident morphological alterations were observed in the midgut epithelium of mosquitoes treated with DTT, except some loss of mucus continuity when compared to untreated controls (Fig. 2b).

**Fig 3:**
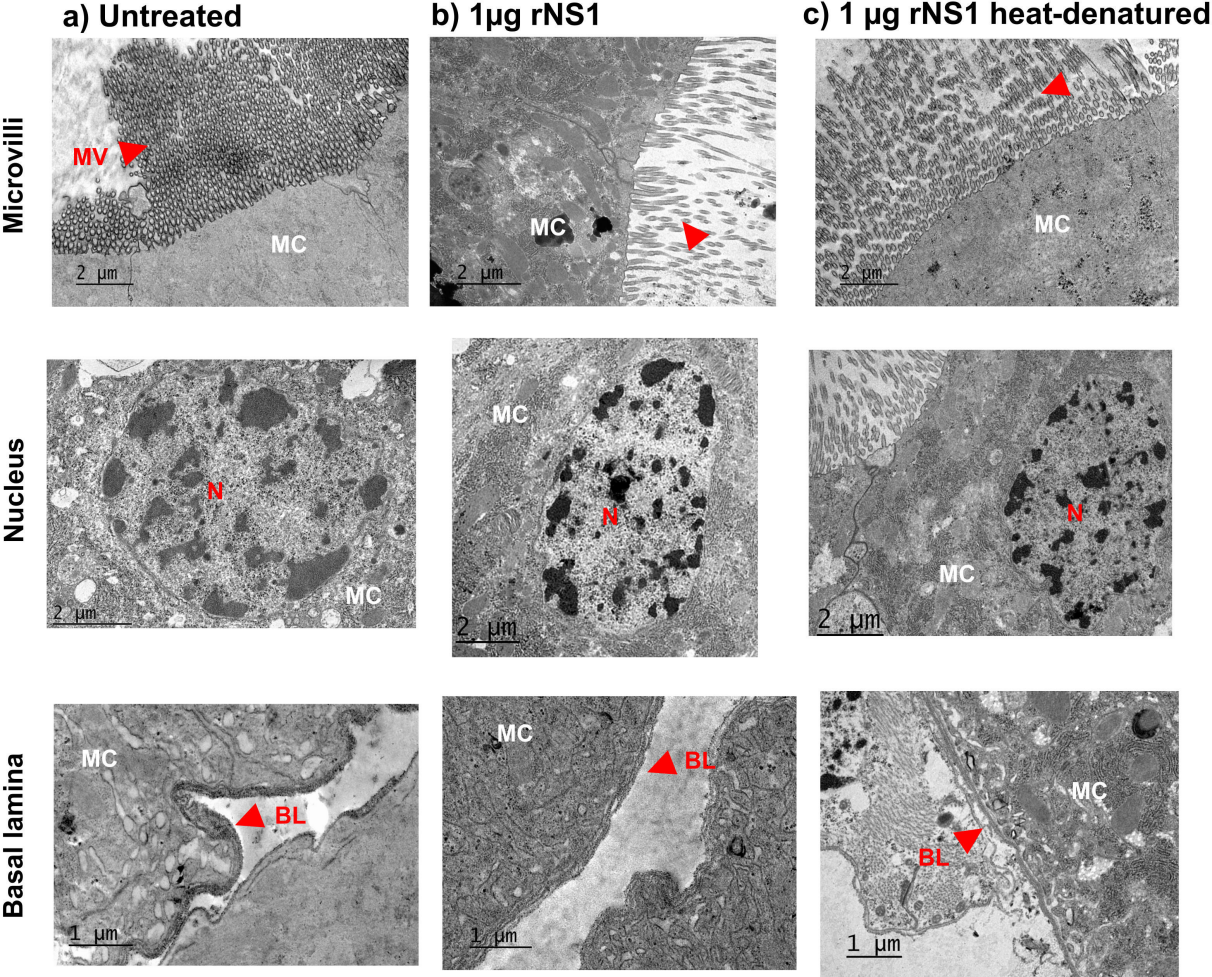
TEM analysis of the midgut epithelial cells of *Aedes aegypti* exposed to rNS1 protein. The red arrows indicate the zone of microvilli. Microvilli in the apical zone of the midgut epithelial cells of the untreated group were numerous and appeared undamaged (**a**); in the presence of the rNS1 protein, there appeared to be a reduction in the number and length of the microvilli (**b**), a damage that was reversed when the protein was denatured by heat (**c**). No other changes were observed in cell morphology, nor the shape of the nucleus and basal lamina. MC: midgut epithelial cells, MV: microvilli, N: nucleus, BL: basal lamina. Five midguts of female mosquitoes per treatment were examined with reproducible results.

The cell microvillus alterations were confirmed by TEM. Ultrastructural analysis of the mosquito midgut showed a reduction in the abundance of microvilli only in the rNS1-fed mosquitoes (Fig. 3a through c). No morphological changes were evident in the cell nuclei (Fig. 3d through f) or in the basal lamina of the examined midgut epithelial cells from all groups (Fig. 3g through i).

### NS1 induces changes in the paracellular permeability of midgut epithelium

**Fig 4:**
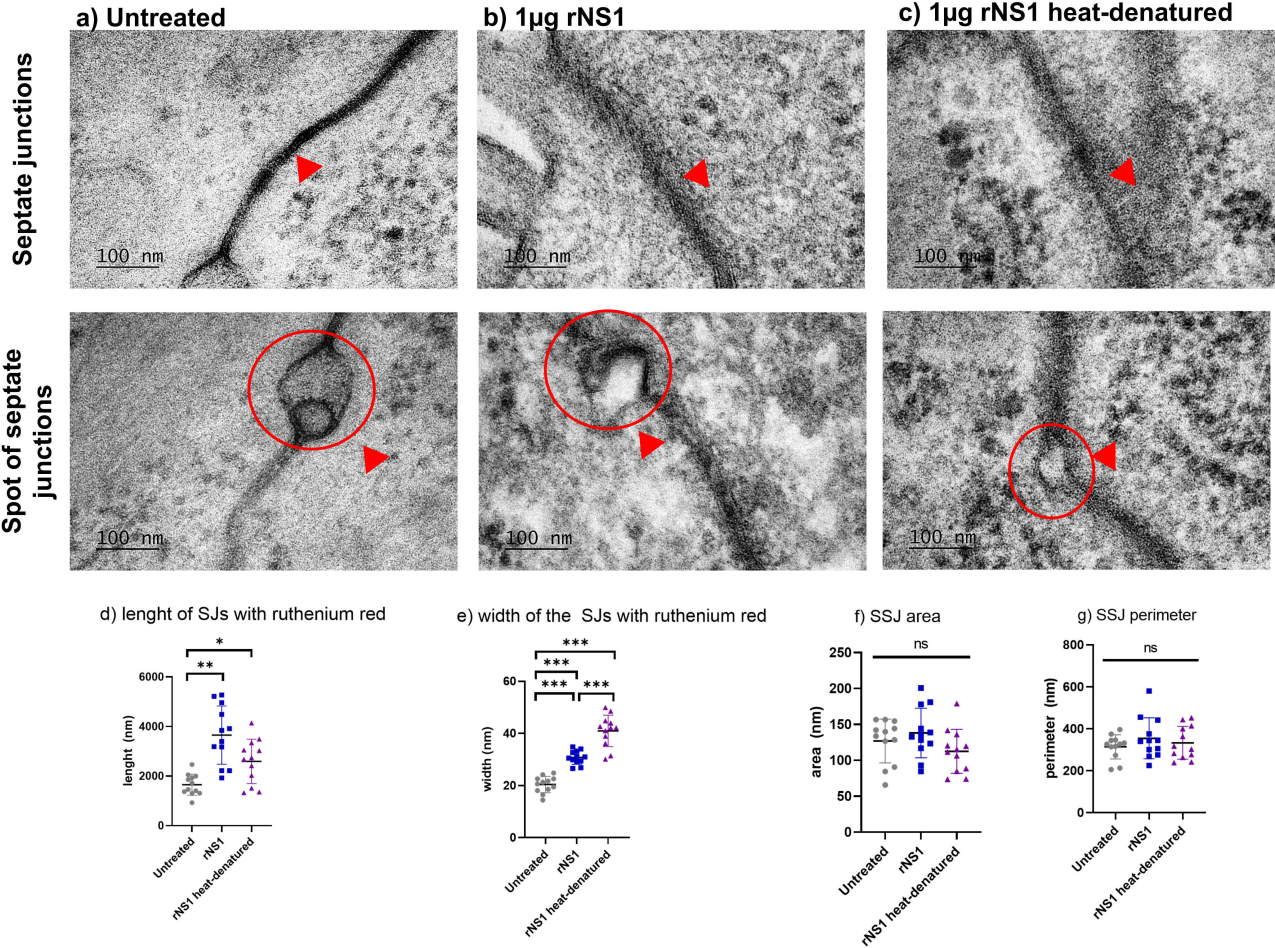
Qualitative changes in the SJs and spot of septate junctions (SSJs) of *Aedes aegypti* exposed to rNS1 protein. The spot is indicated by surrounding it with a red circle and pointing at it with red arrows. In untreated mosquitoes (**a**), the ruthenium red (red arrow) is incorporated into the SJ but is retained in the septum. The SSJ remained intact. When the mosquitoes ingested rNS1, ruthenium red emerged from the SJ septum, and SSJs lost their shape (**b**). Denaturation by heat of the rNS1 did not prevent ruthenium red from leaving the septum, but no changes in the SSJ were observed (**c**). Measurements were made using ImageJ. The highest ruthenium red incorporation was observed in the presence of rNS1 (**d**). The width of the septa when ruthenium red was incorporated. The septa were wider with NS1 compared to the untreated condition; unexpectedly, heat-denatured rNS1 provoked the widest septa (**e**). No changes were observed in areas (**f**) and perimeter (**g**). Three measurements were performed for each independent experiment (*n* = 12 in total). Images were analyzed using Fiji/ImageJ software. The * above icons indicate significant differences between conditions. ANOVA/Tukey test, *P < 0.05; **P < 0.01; ***P < 0.001. Scale bar: 100 nm. ns, no significant difference.

To further investigate midgut changes caused by rNS1 protein, cell-to-cell junctions (CJs) and SJs integrity were evaluated using ruthenium red as an indicator of altered paracellular permeability. Alteration indicators include mainly how far the ruthenium red is incorporated and the width of the septa, but also morphological or perimeter and area variations in the spots located at the SJ or area width where ruthenium red is not intercalated (Fig. S4 and S5). Ruthenium red intercalation at the CJ was observed in all conditions (Fig. 4a through c). However, the length of dye penetration was significantly longer in the rNS1 compared with the untreated midguts or treated with heat-denatured protein (Fig. 4d). Channel width was also larger in the samples with rNS1 than in controls, albeit the width of these channels was even greater in the NS1 heat-denatured condition than in the other conditions (Fig. 4e).

**Fig 5:**
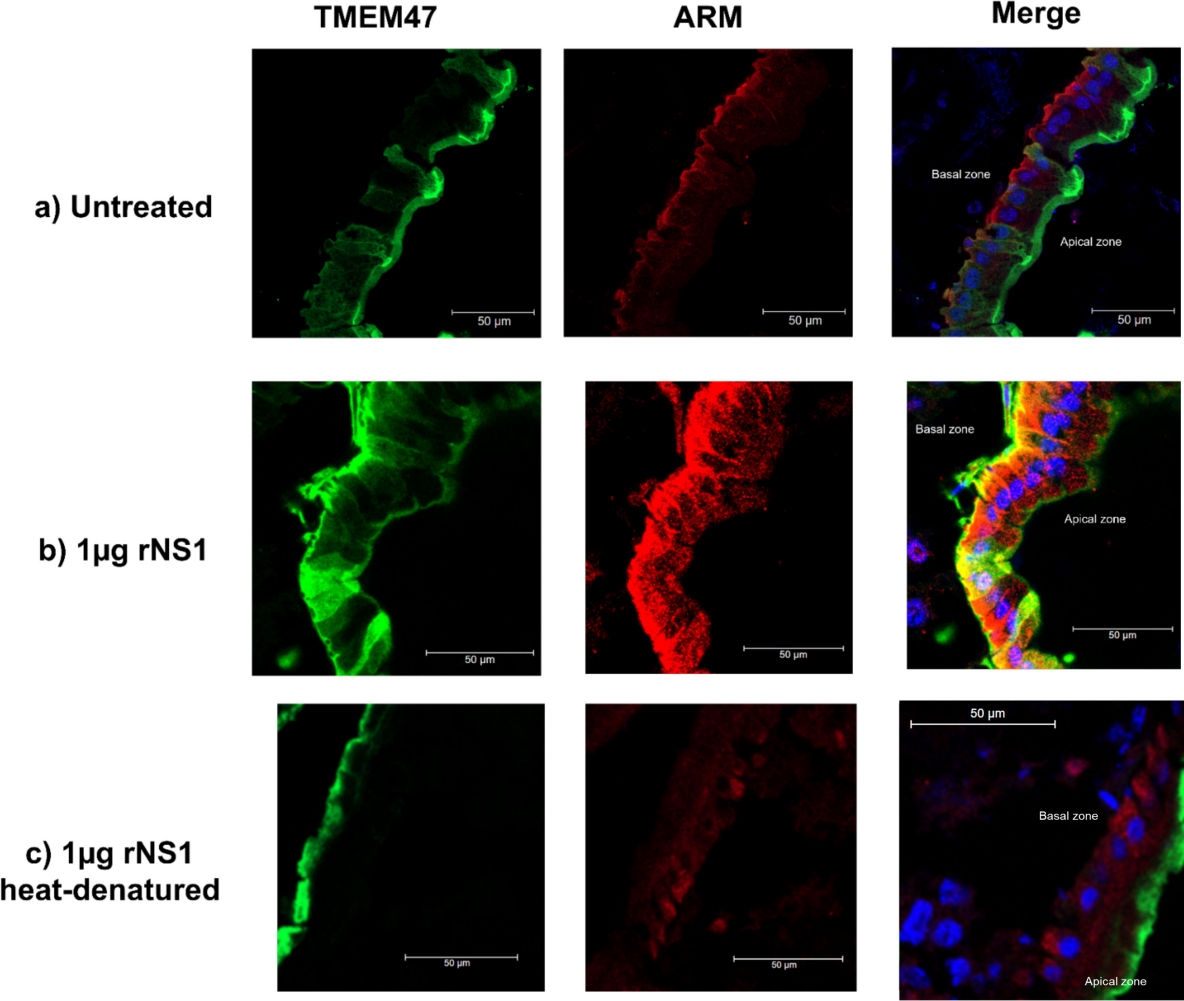
Confocal immunofluorescence microscopy analysis of TMEM47 (green) and ARM (red) proteins in transverse cryosections of *Aedes aegypti* midguts. (**a**) In untreated midguts, TMEM47 localizes at the apical region of the epithelial cells while ARM is present at the basal region. (**b**) rNS1 treatment leads to TMEM47 relocalization toward both apical and basal compartments, whereas ARM is primarily redistributed to the cytoplasmic compartment and nuclei. (**c**) Denatured rNS1 does not alter the typical localization of either TMEM47 or ARM. Nuclei were counterstained with DAPI (blue). Three abdomens of female mosquitoes per treatment were examined with reproducible results. Scale bar: 50 µm. *n* ≥ 3.

Sac-like structures, called spots SJ (SSJ), characterized as intercellular cavities or gaps between CJs, were also examined (38, 39). There was a loss of integrity of these spots in the presence of rNS1 protein compared to untreated midguts (Fig. 4a and b), and their morphology was unaffected when NS1 was denatured (Fig. 4c). None of the treatments significantly changed the area or the perimeter of the SSJ (Fig. 4d and e). No changes were observed in the regions of the SJ where ruthenium red was not intercalated (Fig. S5a through c); however, the width of the septa was altered, being widest in the presence of rNS1 protein (Fig. S5d). Altogether, these ultrastructural alterations support the idea that rNS1 alters the integrity of the mosquito midgut epithelial barrier by remodeling of SJs.

### NS1 induces SJ protein delocalization in midgut epithelium

The integrity of the SJ was also analyzed by confocal microscopy. Initially, an *in silico* comparative analysis using available databases was carried out to find potential *Ae. aegypti* proteins involved in the formation and maintenance of SJ. Two candidate proteins were chosen based on their similarity to known CJ complex components in other organisms: AAEL001447 (TMEM47), a claudin-like transmembrane protein, and AAEL002887 (ARM), an ortholog of the Armadillo segment polarity protein. Both proteins are related to CJ proteins found in vertebrates (claudins and β-catenins, respectively). Phylogenetic analysis and domain prediction confirmed the relationship with these types of protein (Fig. S6). These two candidate proteins contain antigenic domains homologous to those recognized by antibodies directed to claudin-1 and β-catenin of vertebrates. Therefore, we analyzed the localization of these candidates in the mosquito midguts exposed to rNS1 protein.

**Fig 6:**
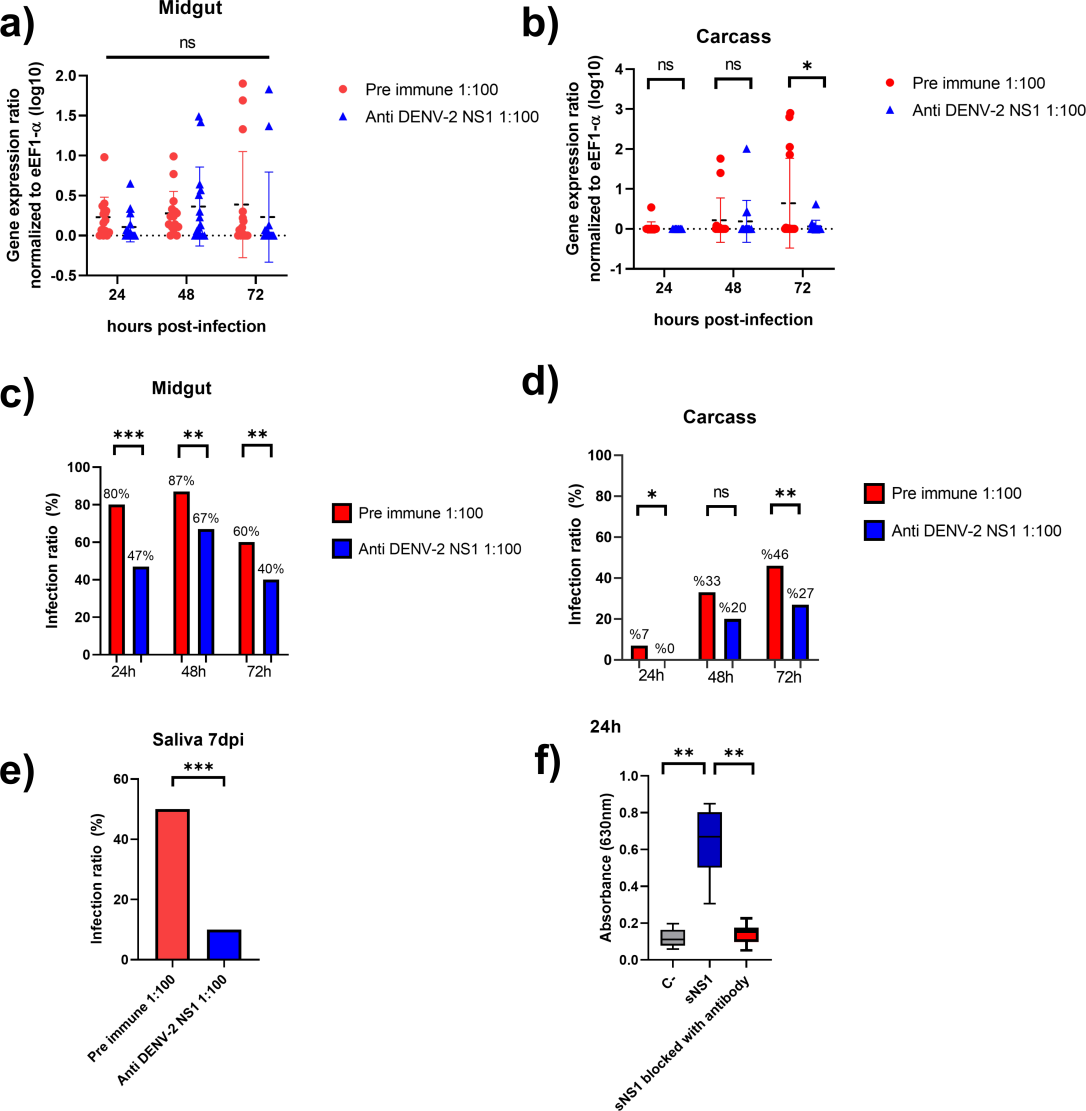
NS1 protein promotes virus dissemination to secondary tissues in *Ae. aegypti*. Female mosquitoes were orally infected with DENV-2 in combination with pre-immune serum (red) or anti-NS1 antibodies (blue). Relative quantification of viral genomes was evaluated at 24, 48, and 72 h post-infection in midguts (**a**) and carcasses (**b**). The infective ratio was evaluated at 24, 48, and 72 h post-infection in midguts (**c**) and carcasses (**d**). Virus presence was evaluated in the salivary gland at 7 days post-infection (**e**). Quantification of dye outflow at 24 hpt in mosquitoes exposed to infected cell supernatants with secreted NS1 (sNS1) treated with pre- or hyperimmune sera to NS1 (**f**). The untreated control condition is the same as shown in Fig. 1 at 24 hpi (**e**). *P < 0.05, **P < 0.01, ***P < 0.001. *n* ≥ 3.

In non-treated midguts, TMEM47 is specifically localized in the apical region and ARM in the basal region of the epithelial cell (Fig. 5a). The treatment with rNS1 protein induced a delocalization of TMEM47 from the apical to the basal region. Interestingly, the localization of the ARM protein was also altered, delocalizing mainly to the cytoplasm and nuclei (Fig. 5b). None of these changes were observed when the rNS1 protein was denatured (Fig. 5c). These results indicate that NS1 alters the location of key component proteins of SJs.

It has been reported using mammalian host-derived cells that the alteration in tight junction proteins by NS1 is associated with changes in the expression of several metalloproteinases (40–42). Thus, the expression levels of two metalloproteinase genes, Aemmp1 and Aemmp2, involved in basal lamina degradation in mosquitoes during CHIKV infection (23, 43, 44), were evaluated by qRT-PCR in midguts exposed to rNS1. A significant decrease in Aemmp1 expression was observed in mosquitoes treated with rNS1 at 24 h post-feeding (Fig. S7). The rNS1 heat-denatured produced a non-significant reduction in Aemmp1 expression. A decrease in Aemmp2 messenger expression was observed in the presence of DENV rNS1, either heat-denatured or not, at 24 and 48 hpf. However, Aemmp2 expression was recovered at 48 hpf in samples treated with heat-denatured rNS1 (Fig. S7). These results suggest that inhibition of MMP expression occurs along with the delocalization of TMEM47 and ARM proteins.

**Fig 7:**
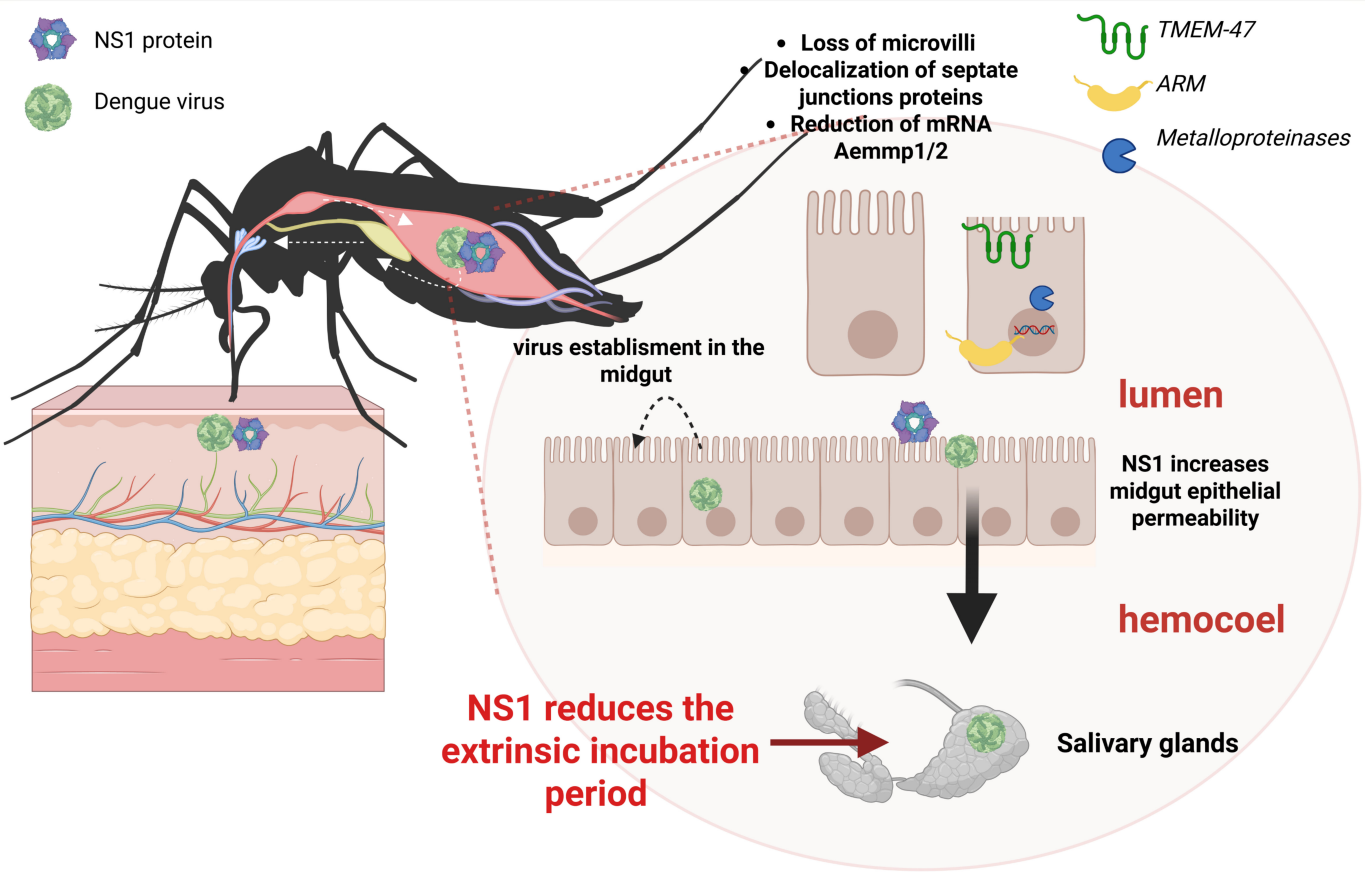
Scheme summarizing the effects of NS1 on the intestinal midgut barrier and on virus dissemination in the mosquito. Upon exposure to NS1, enterocytes show loss of microvilli, delocalization of SJ proteins (*TMEM-47* and *ARM),* and reduced expression of *AeMMP1* and *AeMMP2* genes. These alterations increase epithelial permeability, facilitating early viral escape into the hemocoel and rapid dissemination to the salivary glands. Overall, NS1 reduces the extrinsic incubation period, potentially enhancing mosquito transmission efficiency.

### NS1 protein promotes DENV dissemination in *Ae. aegypti*

Given that DENV-2 NS1 alters the midgut epithelial barrier by increasing paracellular permeability and disrupting the SJ proteins, we hypothesized that these alterations may facilitate viral dissemination to secondary tissues. To evaluate the role of NS1 in DENV-2 dissemination, we compared viral spread in mosquitoes where NS1 function was either intact or inhibited. Supernatants collected from DENV-infected BHK-21 cells were pre-incubated with pre-immune serum or hyperimmune anti-NS1 serum (1:100 for both conditions) before feeding the female mosquitoes. Anti-NS1 antibodies can block NS1 activity by inhibiting binding to cells or disrupting the hexameric structure (45). The capacity of these supernatants to cause midgut permeability was tested in intestine permeability assays (Fig. 6f). In addition, the NS1 presence in the supernatants was tested with a commercial ELISA kit (data not shown) and dot-blot assays (Fig. S3), and virus load by qRT-PCR (data not shown). Viral dissemination was assessed in the midgut, carcass, and saliva across infection time points by qRT-PCR.

Relative viral load analysis revealed no significant differences between treatments in the midgut (Fig. 6a) or carcass (Fig. 6b), except at 72 hpi in carcasses, where mosquitoes fed with supernatants incubated with pre-immune serum exhibited higher viral loads (*P* < 0.05). Nonetheless, infection ratios in the midgut showed significant differences starting at 24 hpi, with the unblocked NS1 group reaching an 80% infection rate (*P* < 0.001). This trend persisted at 48 and 72 hpi (*P* < 0.01) (Fig. 6c). Similarly, in the carcass, infection in the unblocked NS1 group began increasing from 24 hpi, reaching 47% at 72 hpi, compared to only 27% in the NS1-blocked group (*P* < 0.05) (Fig. 6d).

These results suggest that NS1 facilitates the dissemination of the virus from the midgut to secondary tissues, likely contributing to a shortened extrinsic incubation period. To further investigate the dissemination process, saliva samples were analyzed at 7 days post-infection. Mosquitoes exposed to unblocked NS1 showed a significantly higher infective ratio (46%) compared to the NS1-blocked group (27%) (*P* < 0.01) (Fig. 6e), indicating that NS1 promotes early viral escape from the midgut, enhancing vector competence.

## DISCUSSION

The DENV NS1 protein circulates in the blood of patients during the acute phase of disease at concentrations that reach micrograms per microliter (46). Thus, it is expected to be ingested along with the virions during a mosquito bite. Previous evidence indicated that ingested NS1 favors DENV infection in the mosquito by tuning down mosquito innate immunity (24). In this study, we demonstrate that NS1 enhances mosquito infection and viral dissemination by a novel mechanism, the disruption of the midgut epithelial barrier of *Ae. aegypti*. Using a dye leakage assay, we found that rNS1 increases midgut permeability in a dose-dependent and transient manner. This dye-based assay has been successfully employed in other models, such as *Drosophila melanogaster* (29, 30) and *Anopheles gambiae* (47), where disruption of the midgut barrier leads to dye leakage into the hemocoel. Histological and ultrastructural analyses revealed that rNS1 exposure leads to loss of microvilli and morphological and functional alterations in SJs, whereas immunofluorescence assays showed that these alterations go along with the delocalization of two key junctional proteins, TMEM47 and ARM. These epithelial alterations correlated with a higher infection ratio in the midgut and secondary tissues and an accelerated presence of DENV in mosquito salivary glands. Heat denaturation of NS1 or incubation with anti-NS1 antibodies reduced these effects, suggesting that NS1 acts as a mediator of epithelial permeability, virus dissemination, and vector competence. Recently, the participation of infected hemocytes in DENV and ZIKV dissemination to peripheral mosquito tissues, including ovaries and salivary glands, has been reported (48). By disrupting the intestinal barrier and allowing ingested virions to reach the hemolymph, NS1 may be directly contributing to facilitating hemocyte infection.

Similar effects of DENV NS1 have been reported on vertebrate vascular endothelium, where secreted NS1 disrupts the endothelial glycocalyx and degrades tight and adherent junction proteins, leading to hyperpermeability (10). In human endothelial cells, NS1 recruits host metalloproteinase 9 (MMP-9) to degrade β-catenin, ZO-1, and ZO-2, further compromising cell–cell adhesion (40–42). The present work extends this paradigm to the mosquito midgut, showing that a similar junctional disruption mechanism may underlie both the vascular leak in the vertebrate host and midgut barrier dysfunction in the vector.

The morphological alterations observed by TEM in the midgut microvilli may unveil a mechanism by which the NS1 protein facilitates access to its target cells, as microvilli are known to act as physical barriers that hinder pathogen interaction with epithelial surfaces (32, 49). The reduction or disorganization of these structures could therefore reflect an NS1-driven mechanism to weaken this first line of defense. Furthermore, the increased penetration of ruthenium red between epithelial cells suggests that NS1 disrupts SJs, since intact junctions normally prevent the intercalation of this tracer (32). Also, the NS1 alters the morphology of the SSJs without changing their overall area or perimeter. The altered ultrastructure we observed supports the notion that NS1 compromises epithelial integrity, thereby enhancing paracellular permeability (40, 41).

These findings are in line with previous reports showing that ultrastructural disruptions of the basal lamina in *Ae. aegypti* are associated with increased arboviral dissemination (20, 22, 44). Secondary blood meal induces transient basal lamina damage that facilitates DENV escape from the midgut (19). Similarly, TEM studies of CHIKV infection have documented localized basal lamina breaks associated with enhanced viral spread (44). These findings suggest that NS1-induced epithelial remodeling may act in synergy with natural physiological processes or viral strategies to promote early systemic infection in the mosquito.

In addition, the inhibition of components involved in basal lamina repair, such as MMPs, was also observed. Direct inhibition of MMPs in an *Ae. aegypti*-CHIKV model failed to block the spread of this virus (44). In other models, the evidence suggests that MMPs are essential for both the degradation and repair of the extracellular matrix. When MMP expression is reduced, there is a slowing/defect in healing, a failure of remodeling, and problems with morphogenesis (50, 51). Thus, interfering with the degradation/restoration dynamics of the basement membrane can affect virus dissemination.

Our findings also revealed that rNS1 caused delocalization of key SJ components, namely TMEM47 (claudin-like) and ARM (β-catenin ortholog), associated with SJ integrity and barrier functions in the mosquito midgut epithelium. These results are consistent with the behavior of smooth SJ in *Drosophila*, where proteins such as Mesh, Ssk, and Tsp2A are critical for barrier function and display mutual dependency for proper membrane localization. Disruption of any of these components leads to junctional breakdown, cytoplasmic mislocalization, and increased paracellular permeability (38, 52). TMEM47, in particular, belongs to the claudin superfamily and has been proposed as a functional analog of Ssk or claudin-like proteins in mosquitoes, while ARM shares structural features with β-catenin and may be involved in anchoring cytoplasmic complexes at the junction. These changes also mirror phenotypes observed in genetic knockdown models of SSJ proteins in *Drosophila*, where similar relocalization and leakage phenotypes are reported (53). Thus, the observed altered localization patterns are therefore indicative of NS1-induced disassembly of the SJ complex, compromising the epithelial integrity and likely facilitating paracellular viral dissemination. The ingested NS1 most likely causes these alterations, since they are observed at times too early to expect significant endogenous production and secretion of NS1, as suggested by our own (Fig. S3) and others’ observations (12). Despite these new findings, several limitations must be considered. First, we used rNS1 added externally, which may not fully reflect the dynamics or state of NS1 circulating in patients’ sera, where NS1 has also been reported to circulate in complexes with HDL and LDL (54, 55). Second, rNS1 from DENV-2 was primarily used, and although NS1 is highly conserved (56), our preliminary data with rNS1 from ZIKV suggest that the observations are not specific to DENV, additional confirmation with other orthoflavivirus NS1 is required. Third, the Rockefeller strain of *A. aegypti*, highly susceptible to DENV, was used, and virus dissemination may differ in natural mosquito populations.

The midgut is the first tissue where the virus interacts with the vector (11, 57, 58). Therefore, overcoming the midgut infection barrier is essential for viral establishment. Our results taken together indicate that NS1 (i) directly contributed to the early establishment of DENV in the midgut by altering epithelial permeability and junctional remodeling and subversion of tissue repair. This novel mechanical function will pair with physiological stimuli such as blood feeding, which by itself induces microfractures in the midgut epithelium, and with the immunomodulatory capacity reported for NS1. Previous studies have reported that DENV and ZIKV NS1 present in the blood meal can inhibit the JAK-STAT pathway in *Ae. aegypti* midgut cells, thereby favoring viral establishment (24, 59). It will be worth evaluating whether NS1 may also participate in other cellular processes, such as apoptosis or cytoskeletal remodeling, mechanisms tightly linked to SJ integrity, which have been explored in the *Ae. aegypti-DENV* model, but not in the context of NS1 direct involvement (60–62), facilitates faster viral escape from the midgut into secondary tissues (e.g., carcass) and ultimately the salivary glands. In this regard, the contribution of endogenously synthesized NS1 needs to be evaluated. Mosquito cells are known to secrete NS1 (63, 64), but its function may vary depending on the tissue type, such as fat body or hemocytes. Figure 7 summarizes the main known functions of NS1 in mosquitoes. Finally, these findings suggest that NS1 reduces the extrinsic incubation period and enhances vector competence. Thus, strategies targeting NS1 could reduce the vector competence to DENV infection, offering a novel venue for dengue control.

## SUPPLEMENTAL MATERIAL

Figure S1 mbio.03173-25-s0001.pdf Commercially acquired recombinant NS1 proteins of DENV and ZIKV.

Figure S2 mbio.03173-25-s0002.pdf Experimental conditions used to evaluate the role of native and denatured NS1 ZIKV in modulating epithelial permeability.

Figure S3 mbio.03173-25-s0003.pdf Detection of NS1 protein by dot-blot.

Figure S4 mbio.03173-25-s0004.pdf Incorporation of ruthenium red.

Figure S5 mbio.03173-25-s0005.pdf Changes in the zone where the ruthenium red is not intercalated.

Figure S6 mbio.03173-25-s0006.pdf Identification of two *Ae. aegypti* proteins belonging to the protein complex of the septate junctions.

Figure S7 mbio.03173-25-s0007.pdf Midgut gene expression of metalloproteases *Aemmp1* and *Aemmp2*.

Supplemental legends mbio.03173-25-s0008.pdf Legends to supplemental figures.

Supplemental table mbio.03173-25-s0009.pdf Table S1.

ASM does not own the copyrights to Supplemental Material that may be linked to, or accessed through, an article. The authors have granted ASM a non-exclusive, world-wide license to publish the Supplemental Material files. Please contact the corresponding author directly for reuse.
