# Supplementary figures and images for: The dengue virus NS1 protein alters Aedes aegypti midgut permeability and favors virus dissemination
Source: mBio. 2026 Jan 13;17(2):e03173-25. doi: 10.1128/mbio.03173-25 (PMC12892943; doi:10.1128/mbio.03173-25)

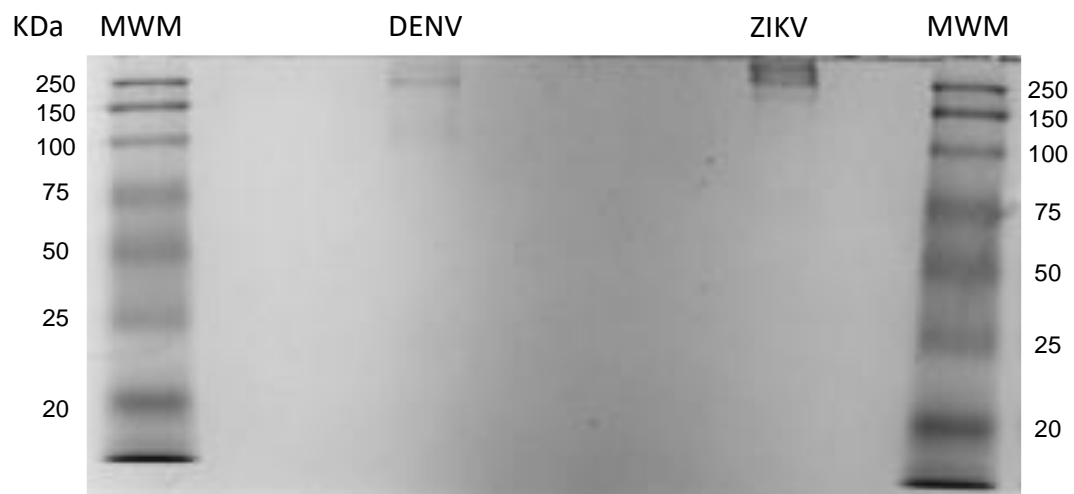

**Supplemental Figure 1**

Supplement: Figure S1 — Commercially acquired recombinant NS1 proteins of DENV and ZIKV. [file mbio.03173-25-s0001.pdf]

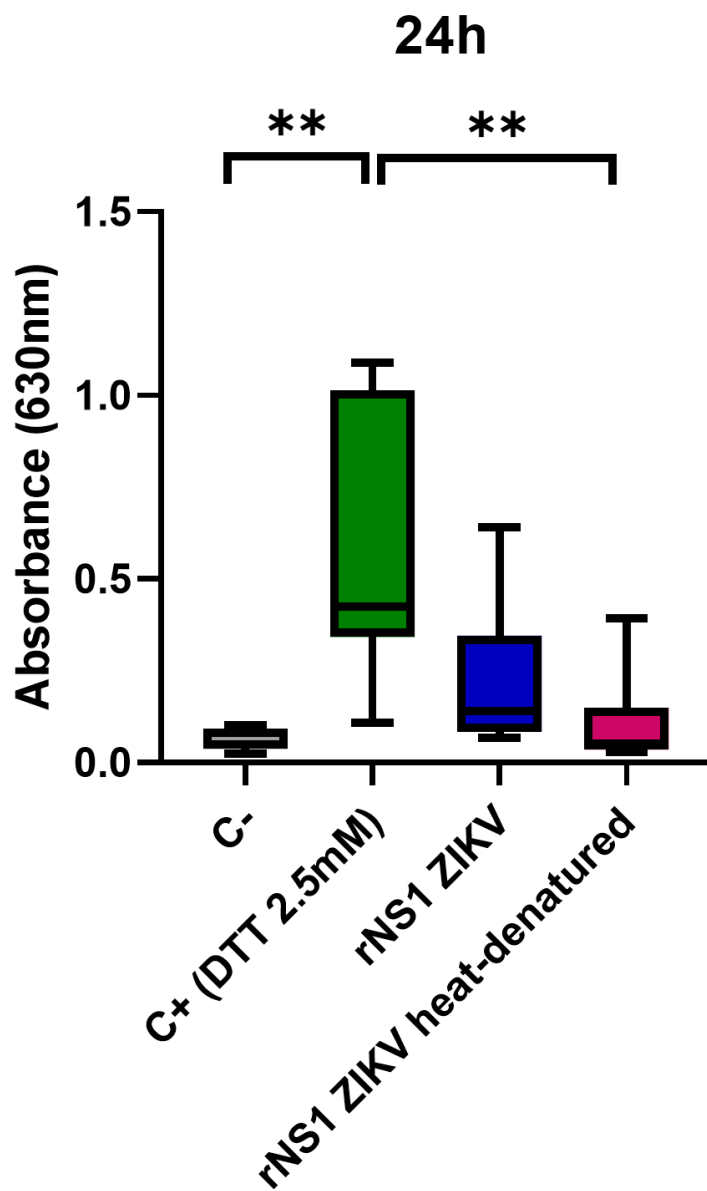

Supplemental Figure 2

Supplement: Figure S2 — Experimental conditions used to evaluate the role of native and denatured NS1 ZIKV in modulating epithelial permeability. [file mbio.03173-25-s0002.pdf]

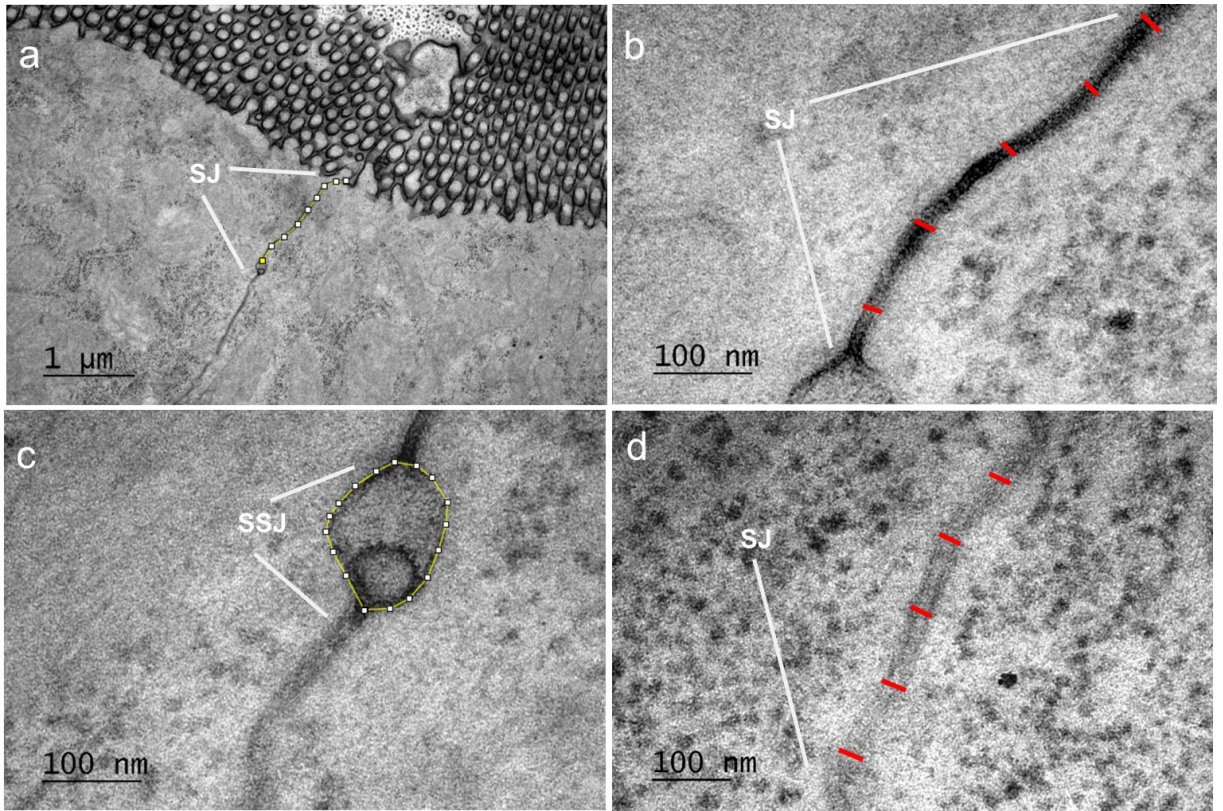

**Supplemental Figure 4**

Supplement: Figure S4 — Incorporation of ruthenium red. [file mbio.03173-25-s0004.pdf]

a) Untreated

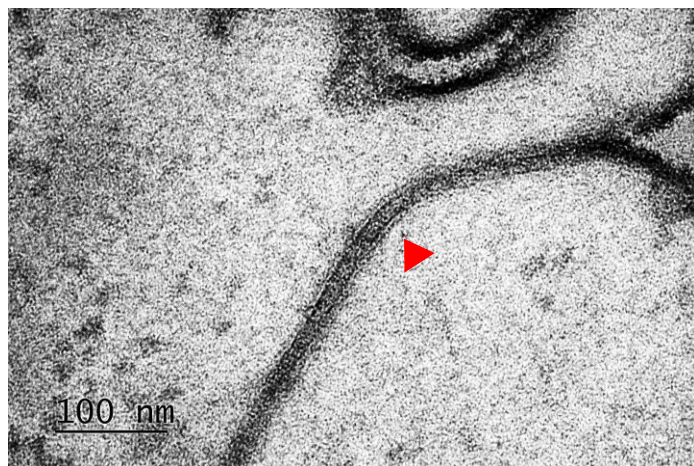

b) 1 $\mu$ g rNS1

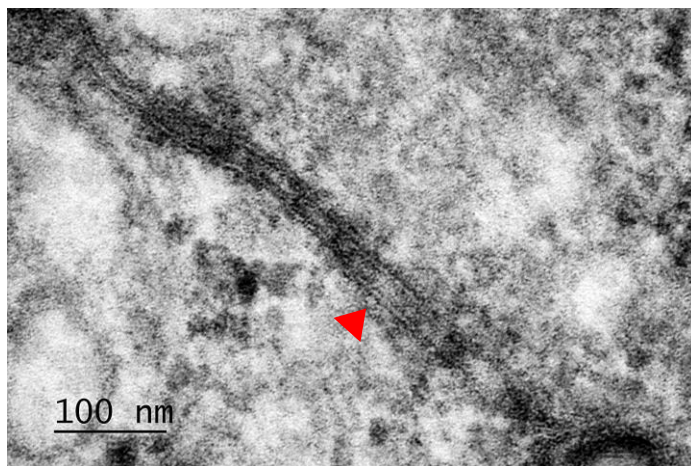

c) 1 $\mu$ g rNS1 heat-denatured

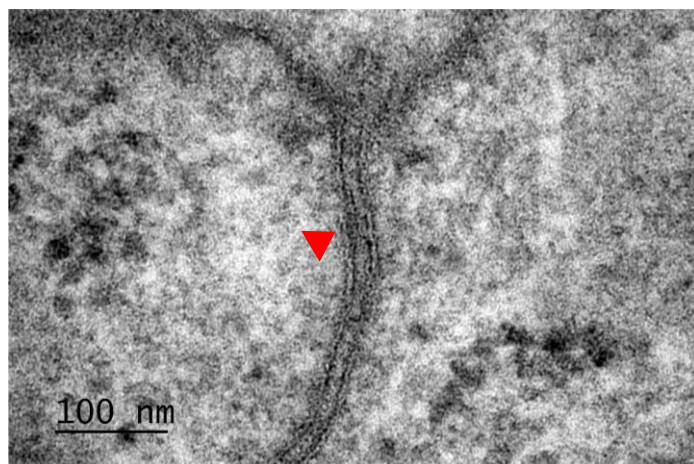

d) width of the SJs

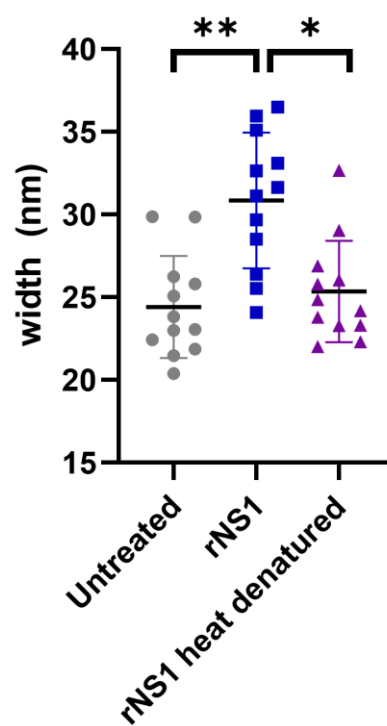

Supplemental Figure 5

Supplement: Figure S5 — Changes in the zone where the ruthenium red is not intercalated. [file mbio.03173-25-s0005.pdf]

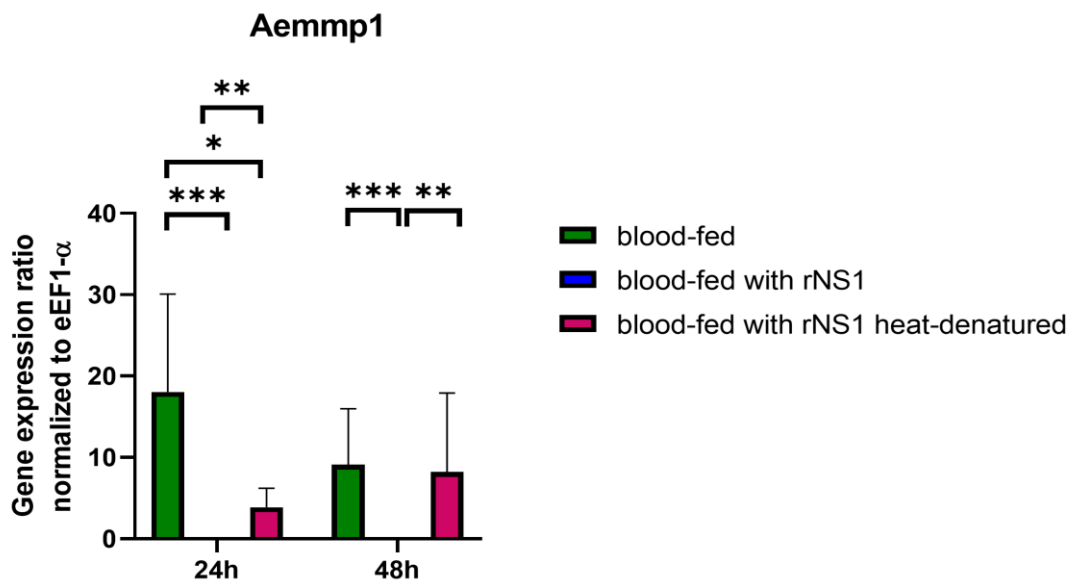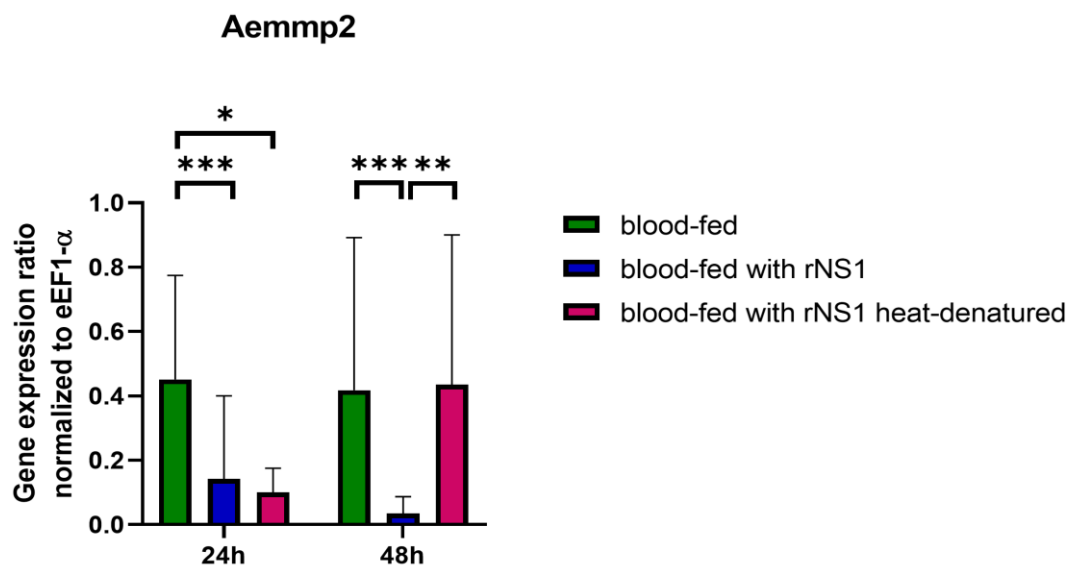

Supplementary Figure 7

Supplement: Figure S7 — Midgut gene expression of metalloproteases Aemmp1 and Aemmp2. [file mbio.03173-25-s0007.pdf]
