# Supplementary material for: The dengue virus NS1 protein alters Aedes aegypti midgut permeability and favors virus dissemination
Source: mBio. 2026 Jan 13;17(2):e03173-25. doi: 10.1128/mbio.03173-25 (PMC12892943; doi:10.1128/mbio.03173-25)
Supplement: Figure S3 — Detection of NS1 protein by dot-blot. [file mbio.03173-25-s0003.pdf]

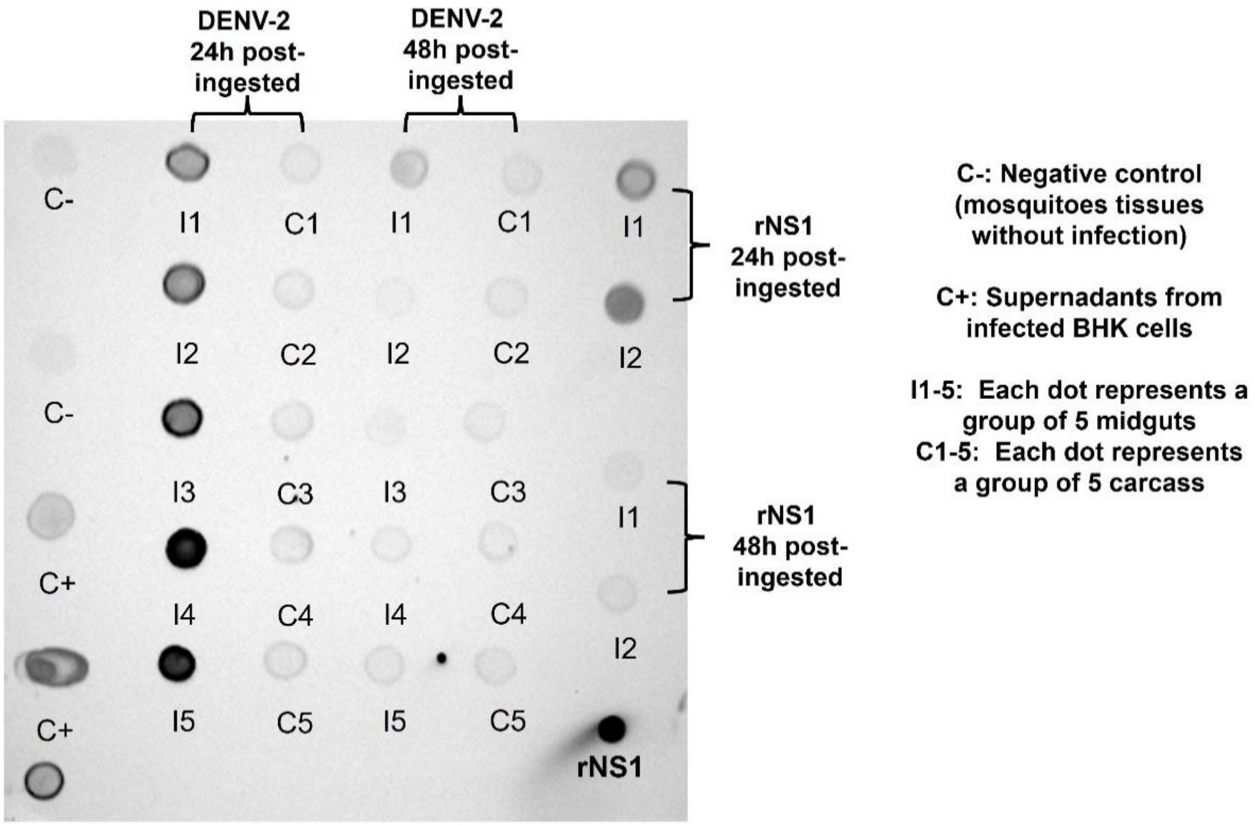

Supplemental Figure 3
