# Supplementary material for: The dengue virus NS1 protein alters Aedes aegypti midgut permeability and favors virus dissemination
Source: mBio. 2026 Jan 13;17(2):e03173-25. doi: 10.1128/mbio.03173-25 (PMC12892943; doi:10.1128/mbio.03173-25)
Supplement: Figure S6 — Identification of two Ae. aegypti proteins belonging to the protein complex of the septate junctions. [file mbio.03173-25-s0006.pdf]

A)

AAEL001447 transmembrane protein 47

>XP_021700801.1 transmembrane protein 47 isoform X1 [Aedes aegypti]
MSKALTAKSDEYYHRQLGLSGIQSSFDGDSLPDYDEFDSESVTLEYFKESTKRGPSPDKMAPTTTIETVTITRPLK
VIAFICGVIVGILMILALTSTDWLMAESWRQGLFVHCIEEDYSPPLPFNLDPAGCYASRDVTYIKATAGLCIITLVTDV
IATILTGLGLNTQDHNRKYKFYRVAVLVMMVALISLLVALILYPVCFAAELNLDPHFIAGNRPVWEFGWAYGVGWGA
AIFLFGAVVLLLCDKESEEIYYKERKIVHDASMRD

select all   *15 sequences selected*   GenPept   Graphics   Distance tree of results   Multiple alignment   MSA Viewer

	Description	Scientific Name	Max Score	Total Score	Query Cover	E value	Per. Ident	Acc. Len	Accession
☑	uncharacterized protein Dmel_CG45049, isoform D [Drosophila melanogaster]	Drosophila melanogaster	375	375	93%	3e-131	77.51%	273	NP_001287471.1
☑	uncharacterized protein Dmel_CG45049, isoform B [Drosophila melanogaster]	Drosophila melanogaster	324	324	78%	7e-112	79.33%	203	NP_001097876.1
☑	transmembrane protein 47 [Mus musculus]	Mus musculus	60.1	60.1	70%	1e-09	25.39%	181	NP_620090.1
☑	transmembrane protein 47, isoform CRA_a, partial [Mus musculus]	Mus musculus	60.8	60.8	70%	2e-09	25.39%	224	EDL29169.1
☑	transmembrane protein 47 [Homo sapiens]	Homo sapiens	58.9	58.9	70%	4e-09	25.39%	181	NP_113630.1

InterProScan Search Result

Accession	Short Name	Name	Source Database	Matches
IPR015664	P53_induced	P53-induced protein	InterPro	100 200
IPR004031	PMP22/EMP/MP20/Claudin	PMP-22/EMP/MP20/Claudin	InterPro	100 200

Phylogenetic tree (FastME)

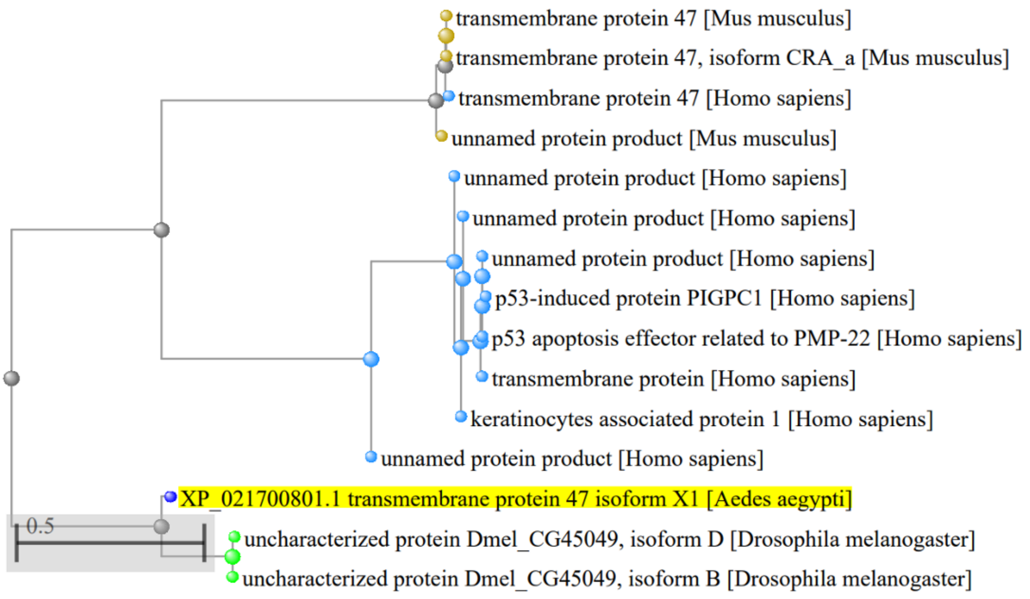

Supplemental Figure 6

B)

AAEL002887 armadillo segment polarity protein

>XP_021700521.1 armadillo segment polarity protein isoform X1 [Aedes aegypti]
MSYQMPQNRTMSHNPYNSSDMPMPSAKEQTLMWQQNSYLGDSGIHSGAVTQVPSLSGKDDDMEDDPLMFDMD
QGFSQNFTQDQVDDMNQQLSQTRSQRVRAAMFPETLEEGIEIPSTQFDPQQPTAVQRLSEPSQMLKHAVVNLINY
QDDADLATRAIPELIKLLNDEDQVVVSQAAMMVHQLSKKEASRHAIMNSPQMVAALVRALSQSNDLETTGAVGTLH
NLSHHRQGLLAIFKSGGIPALVKLLSSPVESVLFYAITTLHNLLLHQDGSKMAVRLAGGLQKMVALLQRNNVKFLAIVT
DCLQILAYGNQESKLIILASTGPSELVRIMRSYDYEKLLWTTSRVLKVLSVCSSNKPAIVEAGGMQALAMHLGNPSQR
LVQNCLWTLRNLSDAATKVDGLETLLSGLVTVLGSSDVNVVTCAAGILSNLTCNNQRNKVTVCQVGGVEALVGTIIN
AGDREEITEPAVCALRHLTSRHPESESAQNIVRNGYGLPVIVKLLNPPSRWPLIKAVIGLIRNLALCPSNAAPLREHGA
IHLLVRLLFKAFQDTQRQRSSVATNGSQPPGAYADGVRMEEIVEGTVGALHILSKEELNRQLIRQQNVISIFVQLLFYN
DIENIQRVAAGVLCELAVDKEVAEMIEAEGATAPLTELLNSANEGVATYAAAVLFKMSEDKSMDYKKRFSSELTTLPVF
RDDTMWNNGELGIGPDLQDILSPDQAYEGLYGQGPPSVHSSHGGRAFQQGYDTLPIDSMQGLEIGGGGNAGPAG
SNPNAGNNPGAGGPPSGQPTSPYAMDMDVGEMDASELTFDHLDVMPSPPQDNNQVAAWYDTDL

select all *100 sequences selected* GenPept Graphics Distance tree of results Multiple alignment MSA Viewer

Description	Scientific Name	Max Score	Total Score	Query Cover	E value	Per. Ident	Acc. Len	Accession
armadillo, isoform F [Drosophila melanogaster]	Drosophila melanogaster	1400	1400	100%	0.0	83.89%	843	NP_001259149.1
armadillo, isoform C [Drosophila melanogaster]	Drosophila melanogaster	1256	1256	86%	0.0	87.07%	721	NP_726775.2
catenin beta-1 isoform 1 [Mus musculus]	Mus musculus	1017	1017	98%	0.0	67.20%	781	NP_001159374.1
unnamed protein product [Mus musculus]	Mus musculus	1017	1017	98%	0.0	67.20%	781	BAB31250.1
catenin beta-1 isoform 2 [Mus musculus]	Mus musculus	1015	1015	98%	0.0	67.20%	780	NP_001418603.1
catenin beta-1 [Homo sapiens]	Homo sapiens	1015	1015	98%	0.0	66.95%	781	BAG70078.1

InterProScan Search Result

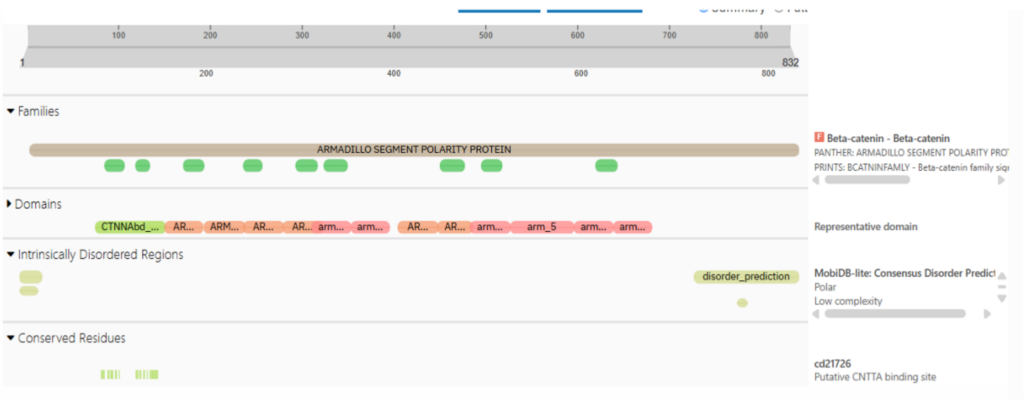

Phylogenetic tree (FastME)

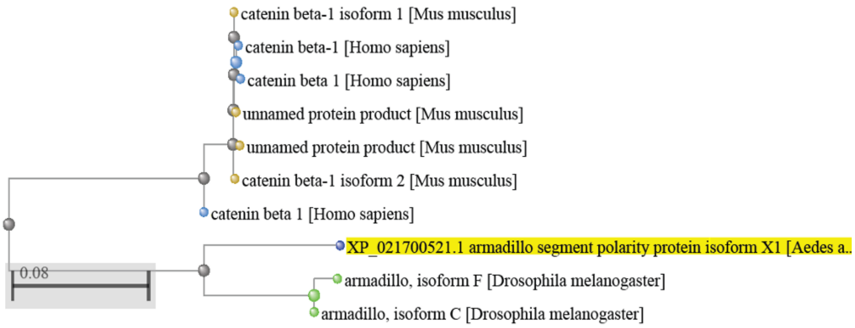

Supplemental Figure 6 cont.
