# Supplementary material for: The dengue virus NS1 protein alters Aedes aegypti midgut permeability and favors virus dissemination
Source: mBio. 2026 Jan 13;17(2):e03173-25. doi: 10.1128/mbio.03173-25 (PMC12892943; doi:10.1128/mbio.03173-25)
Supplement: Supplemental table — Table S1. [file mbio.03173-25-s0009.pdf]

Supplemental Table 1. Primers sequences and gBlock sequence used in this work.

Gene	Forward sequence (5’→3‘)	Reverse sequence (5’→3‘)
DENV	GCTGAAACGCGAGAGAAACC	TCCCTGCTGTTGGTGGGAT
Aemmp1	CAAACAACGTCACAAGCAGAGC	AGCTCTGGAACTCCATGATTGC
Aemmp2	CAACTCGCAACATTTGGAACC	CGTCTTCGTCGAATCTCCAATAA
DENV-2	CAATATGCTGAAACGCGAGA	CAGTTTTAATGGTCCTCGTCC
eEF1α	AGGAATTGCGTCGTGGATAC	GTTCTCTTCGGTCGACTTGC
gblock	ATCGGAAGCTTGCTTAACACAGTTCTAACAGTTTGTTTAGATAGAGA GCAGATCTCTGCAAAAATGAACCAACGAAAAAAGGTGGCTAGACC ACCTTTCAATATGCTGAAACGCGAGAGAAACCGCGTATCAACCCCT CAACGGTTGGTGAAGAGATTCTCGACCGGACTTTTTTCCGGGAAA GGACCCTTACGGATGGTGTTGGCATTCATTACGTTTTTGCGAGTTC TTTCCATCCCACCAACAGCAGGGATTCTGAAAAGATGGGGACAGTT AAAGAAAAACAAGGCCATAAAGATACTCACTGGATTCAGGAAGGAG ATAGGCCGCATGCTGAACATCTTGAATGGTAGAGAGCAGATCTCTG ATGAATAACCAACGAAAAAAGGCGAGAAATACCCCTTTCAATATGCT GAAACGCGAGAGAAACCGCGTGTCGACTGTACAACAGCTGACAAA GAGATTCTCACTTGGAATGCTGCAGGGACGAGGACCATTAAAACT GTTC	
